# Identification of novel Notch target genes in T cell leukaemia

**DOI:** 10.1186/1476-4598-8-35

**Published:** 2009-06-09

**Authors:** Nicholas Chadwick, Leo Zeef, Virginia Portillo, Carl Fennessy, Fiona Warrander, Sarah Hoyle, Anne-Marie Buckle

**Affiliations:** 1 Faculty of Life Sciences, Manchester Interdisciplinary Biocenter, University of Manchester, Manchester M1 7DN, UK; 2 Faculty of Life Sciences, Michael Smith Building, University of Manchester, Manchester M1 7DN, UK

## Abstract

**Background:**

Dysregulated Notch signalling is believed to play an important role in the development and maintenance of T cell leukaemia. At a cellular level, Notch signalling promotes proliferation and inhibits apoptosis of T cell acute lymphoblastic leukaemia (T-ALL) cells. In this study we aimed to identify novel transcriptional targets of Notch signalling in the T-ALL cell line, Jurkat.

**Results:**

RNA was prepared from Jurkat cells retrovirally transduced with an empty vector (GFP-alone) or vectors containing constitutively active forms of Notch (N1ΔE or N3ΔE), and used for Affymetrix microarray analysis. A subset of genes found to be regulated by Notch was chosen for real-time PCR validation and in some cases, validation at the protein level, using several Notch-transduced T-ALL and non-T-ALL leukaemic cell lines. As expected, several known transcriptional target of Notch, such as HES1 and Deltex, were found to be overexpressed in Notch-transduced cells, however, many novel transcriptional targets of Notch signalling were identified using this approach. These included the T cell costimulatory molecule CD28, the anti-apoptotic protein GIMAP5, and inhibitor of DNA binding 1 (1D1).

**Conclusion:**

The identification of such downstream Notch target genes provides insights into the mechanisms of Notch function in T cell leukaemia, and may help identify novel therapeutic targets in this disease.

## Background

Recently, studies have shown that Notch signalling may play a central role in the development of T cell lymphoblastic leukaemia (T-ALL). Since the identification of human Notch1 as a gene involved with a t(7;9)(q34;q34.3) chromosomal translocation in a subset of patients with T-ALL [1], several studies have implicated dysregulated Notch signalling in the aetiology and pathogenesis of T-ALL: Mice transplanted with bone marrow cells transduced with a constitutively active form of Notch1 develop T cell neoplasms [2], while mice transgenic for constitutively active form of Notch3 [3] develop thymic lymphomas. Moreover, Notch3 has been shown to be highly expressed by T-ALL cells and reduced level of Notch signalling was found to correlate with disease remission [4]. More recently, Weng et al. have identified Notch1 gain-of-function mutations in 50% of patients with T-ALL ([5]. These mutations were clustered in the heterodimerisation (HD) and PEST domains of Notch1. HD mutations are thought to enable ligand-independent Notch cleavage and activation, while PEST domain mutations are thought to prolong the half-life of active Notch1. More recently, a new class of Notch1 juxtamembrane expansion mutations have been described in T-ALL which lead to aberrant activation of Notch1 [6]. Interestingly, treatment of T-ALL cell lines with gamma secretase inhibitors (GSIs;) to block Notch activation, inhibited proliferation [7] leading to apoptosis [8], indicating that targeting the Notch signalling pathway may be of therapeutic value in T-ALL. The mechanism of Notch-mediated cell-cycle progression has been shown to be via the direct transcriptional activation of c-myc [9,10], as well as inhibition of PTEN expression [11] and activation of the AKT/PI3K pathway.

Notch signalling has also been shown to inhibit apoptosis in developing thymocytes and in T-ALL cells through a variety of mechanisms: At the protein level, Notch activates the NF-κB pathway [3,12], and activates the PKB/AKT/mTOR pathway-mediated p53 inhibition [13].

While some downstream transcriptional targets of Notch signalling have been identified (for instance the basic helix-loop-helix proteins HES1 [14], HERP1&2 [15]), it is likely that many gene targets of Notch signalling remain to be determined.

Palemero et al. have used microarray analysis to identify novel targets of Notch signalling by treating T-ALL cell lines with GSIs [10]. The cell lines used contained gain-of-function mutations in the Notch1 gene and have over-active Notch signalling [5]. Genes knocked down by GSIs were then further investigated as putative Notch targets, leading to the identification of c-myc as a Notch target gene. A similar approach has also been taken by Weng et al. in a parallel microarray study which also identified c-myc as a target of Notch signalling [9].

We have used an alternative approach by taking a T-ALL cell line (Jurkat) and transducing this cell line with constructs which mimic the gain-of-function Notch1 mutants ("ΔE" constructs which are constitutively activated by gamma secretase). Cells expressing such ectopic Notch constructs were used for Affymetrix microarray analysis to identify putative novel Notch target genes. Following this initial identification, extensive validation of such targets was performed in several T-ALL cell lines using both ectopic Notch expression and Notch knock-down methodologies.

This approach has resulted in the identification of several novel targets of Notch signalling which may play a role in the functional effects of Notch in T-ALL. The identification of such targets may reveal mechanisms by which Notch signalling promotes proliferation and inhibits apoptosis and as such may identify novel targets for therapeutic strategies in T-ALL.

## Methods

### Constructs and cells

N1ΔE (base pairs 5143–7671) and N3ΔE (base pairs 4942–7045) cDNAs were cloned into the bicistronic retroviral vector, pMX-eGFP (a kind gift from T. Kitamura, Tokyo, Japan). pMSCV-DN-MAML1, containing cDNA coding for aa13–74 was a kind gift from J. Aster, Harvard, USA). Retrovirus was produced using the Phoenix amphotropic packaging cell line. Empty pMX or pMSCV vector was used to make the control GFP-alone virus. Cell lines used were Jurkat, CEM, MOLT4, Peer, HPB-ALL, SIL-ALL (T-ALL), Raji (Burkitt's lymphoma), and TF-1 (erythroleukaemia), all cultured in RPMI media containing 10% Fetal Bovine Serum.

Primary CD3+ T cells were isolated from peripheral blood mononuclear cells by flow cytometry and stimulated with 30 ng/ml soluble anti-CD3/anti-CD28 and 100 U/ml IL2 (R&D Systems, Abingdon, UK) in RPMI media containing 10% Fetal Bovine Serum for 72 hrs prior to retroviral transduction.

Retroviral supernatants were used to transduce cells in retronectin-coated tissue culture plates (BioWhittaker, Wokingham, UK). After 48 hrs, GFP+ cells were sorted by flow cytometry and cultured in normal growth medium.

### Affymetrix microarray analysis

GFP+ Jurkat cells transduced with pMX, N1ΔE or N3ΔE were sorted by flow cytometry and total RNA isolated using RNA B (ABgene, Epsom, UK). Four independent transductions were performed to yield 4 sets of total RNA for Affymetrix microarray analysis. RNA quality was checked using the RNA 6000 Nano Assay, and analyzed on an Agilent 2100 Bioanalyser (Agilent Technologies, South Queensferry, UK). RNA was quantified using a Nanodrop ultra-low-volume spectrophotometer (Nanodrop Technologies, Ringmer, UK) and Affymetrix human genome U133A microarrays were used according to the manufacturers' instructions (Affymetrix Inc. High Wycombe, UK). The microarray data has been submitted in MIAME compliant format to Arrayexpress public database (E-MEXP-1744). Microarray data was initially checked for quality using dChip (V2005) software (, [16]). Background correction and quantile normalization were performed using RMA in Bioconductor [17] and differential expression between GFP-alone and Notch constructs were calculated using Cyber-T [18]. Gene lists of differentially expressed genes were controlled for false discovery rate (fdr) errors using the method of QVALUE [19]. Following false discovery rate correction no genes were found to be differentially expressed to a statistically significant level so it was decided to rank by fold change and study the most upregulated genes by qPCR.

### RT-PCR analysis

Total RNA was isolated from GFP+ transduced cell lines or cells treated with gamma secretase inhibitor (GSI IX; Calbiochem) and reverse transcribed to cDNA using the High Capacity cDNA Archive kit (Applied Biosystems, Warrington, UK). Triplicate real-time PCR reactions were performed with PowerSYBR SybrGreen reagents (Applied Biosystems). Fold change in gene expression was determined using the "2^-ddCT^" method using GAPDH as an endogenous control and cDNA from GFP-alone-transduced cells as a calibrator. Primer sequences are shown in Additional file 1.

### GSI washout assay

Jurkat cells were incubated with 10 uM GSI IX for 48 hrs then cells were washed twice with growth media and seeded in growth media in the presence or absence of 20 uM cycloheximide to inhibit protein synthesis. RNA was isolated from cells at various time-points and cDNA used for gene expression analysis.

### ELISA

Cells were seeded at 2 × 10^5^/ml in fresh media and after 4 days, cell supernatants were used for VEGF ELISAs (R&D Systems) according to the manufacturers' instructions.

### Western blotting

Protein extracts from T-ALL cells were used for Western blotting with 1:100 anti-GIMAP5 (anti-IAN4L1 polyclonal antibody; Proteintech, Chicago, USA), anti ID1 (Autogen Bioclear, Calne, UK) or anti b-actin (Sigma, Poole, UK) followed by HRP-conjugated secondary antibodies.

### Luciferase assay

Luciferase assays were performed in HEK293 cells with the reporter construct pGa981–6 [20]. Cells were transfected with reporter contruct and Notch constructs using Fugene6 (Roche, Burgess Hill, UK). After 48 hrs in the presence of DMSO (control) or GSI IX, cells were lysed and luciferase assays performed using standard protocols.

## Results

### Expression of Notch and Validation of Constructs

Since mutations in Notch1 and over-expression of Notch3 have been associated with the development of T-ALL, we focused our attention on these two genes. Quantitative real-time PCR for Notch homologue expression confirmed that Notch1 and Notch3 are the predominantly expressed Notch genes in the Jurkat and CEM T-ALL cell lines (see Additional file 2). In order to identify transcriptional targets of Notch signalling in T-ALL cells, we constructed bicistronic eGFP retroviruses containing the "ΔE" Notch1 or Notch3 cDNA. These constructs express membrane-bound Notch which is constitutively activated by gamma secretase and as such can be inhibited by GSIs. To confirm the activity of these constructs, luciferase assays were performed using a Notch reporter (RBPJ-κ-Luc) with and without GSIs. As can be seen in Additional file 2, both N1ΔE and N3ΔE activated the RBPJ-κ-Luc reporter and this activity could be inhibited by GSIs. However, the activities of Notch intracellular domain (NICD) constructs (which do not require gamma secretase-mediated activation) were not inhibited by GSIs. As well as verifying the activity of these constructs, this result also shows the increased activity of Notch1 compared with Notch3, a finding reported elsewhere [21,22].

### Affymetrix analysis of Notch ΔE-transduced cells

**Figure 1:**
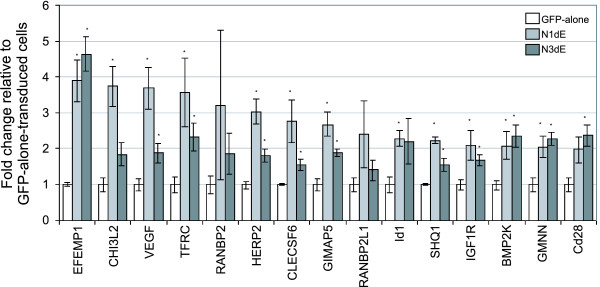
**Affymetrix microarray array analysis of Notch1-transduced Jurkat cells**. Jurkat cells were transduced with GFP-alone, N1ΔE, or N3ΔE retrovirus and after 48 hrs, GFP+ cells were isolated by flow cytometry and total RNA extracted for Affymetrix array analysis. Data presented represents mean of 4 independent experiments. Graphical representation of microarray data showing the 15 genes most upregulated by Notch1. * represents p < 0.05 vesus GFP-alone-transduced cells based on p-values from n = 4 experiments. Using false discovery rate analysis, none of these changes were significant.

GFP-alone, N1ΔE and N3ΔE retroviruses were used to infect the T-ALL Jurkat cell line with a transduction efficiency of approximately 30% and GFP+ cells were sorted by flow cytometry at 48 hrs to generate a pure (> 95%) population of transduced cells for gene expression analysis. This relatively early time-point was used to identify genes directly upregulated by Notch signalling rather those associated with secondary effects of Notch-induced differentiation. Total RNA was made from sorted cells and used for Affymetrix analysis. This procedure was performed in quadruplicate and the Affymetrix data was used to generate mean fold changes in gene expression using the GFP-alone-transduced cells as the calibrator sample. Statistical analysis using false discovery rate correction showed no genes differentially expressed. However, known targets of Notch signalling such as HES1 [23], Notch3 [24], HERP1 and HERP2 [15] were in the top 50 genes ranked by fold change. The 15 genes most upregulated by Notch1 based on analysis of microarray data are shown in figure 1. A high degree of overlap was found with genes upregulated by Notch3 (see Additional file 3). This led us to select the top 10 upregulated genes (as well as CD28; a putative Notch target gene of interest to us) for further analysis. Below we present the results of these validation studies.

### CD28 is a Target of Notch Signalling

**Figure 2:**
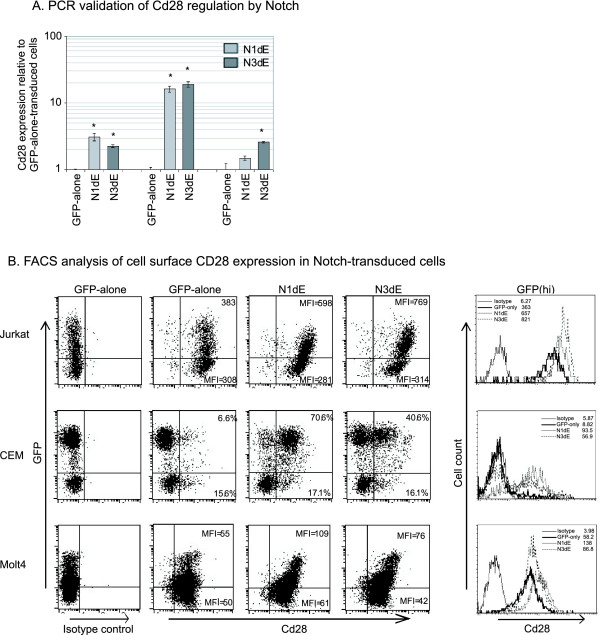
**CD28 expression is upregulated by ectopic Notch signalling**. (A) PCR analysis of CD28 expression in Jurkat, CEM and Molt4 cells transduced with GFP-alone, N1ΔE, or N3ΔE retrovirus. * represents p < 0.05 verus GFP-alone transduced cells. (B) Flow cytometric analysis cell surface CD28 expression using the cells described above. GFP expression is a marker of transduction.

**Figure 3:**
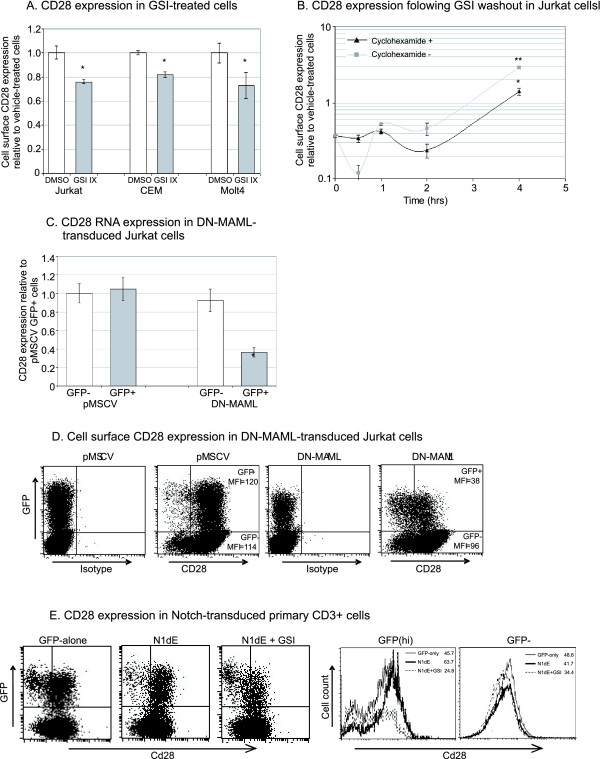
**CD28 expression is downregulated by inhibition of Notch signalling**. (A) T-ALL cell lines were treated with 10 uM GSI IX (or DMSO) for 24 hrs and cell surface CD28 expression analysed by flow cytometry. * represents p < 0.05 verus DMSO-treated cells. (B) GSI washout experiment as described in figure 2 and CD28 mRNA expression analysis in Jurkat cells. * represents p < 0.05 verus cells at the zero timepoint. ** represents p < 0.05 verus cyclohexamide- cells (C) Real-time PCR expression analysis of CD28 using cDNA from GFP- (untransduced) or GFP+ (transduced) Jurkat cells transduced with GFP-alone or N1ΔE retrovirus. * represents p < 0.05 verus GFP-alone transduced cells. (D) Cell surface CD28 expression using the cells described above. (E) CD28 expression in primary CD3+ cells transduced with GFP-alone, N1ΔE, or N1ΔE+GSIs.

CD28 was of interest to us because of its well characterised role in T cell activation [25] and its ability to positively or negatively regulate thymocyte apoptosis ((([26-30]. CD28 was found to be upregulated by both Notch1 and Notch3 in Jurkat cells based on Affymetrix data (figure 1) and we validated this finding by real-time PCR using transduced Jurkat, CEM and Molt4 cells as described above (figure 2.A). We investigated Notch-induced CD28 upregulation at the protein level by flow cytometry. Analysis of GFP-alone- or Notch-transduced Jurkat cells showed a clear upregulation of CD28 expression at the cell surface while untranfected GFP negative cells in the same culture did not show Notch-induced CD28 upregulation (figure 2.B). This effect was seen more clearly in CEM cells where very little basal CD28 expression was seen. The majority of Notch1-transduced cells were CD28 positive, while untransduced cells in the same culture remained negative. Treatment of all T-ALL cell lines with GSIs resulted in a downregulation of cell surface CD28 expression (figure 3.A), showing that endogenous Notch signalling contributes to CD28 expression. This was confirmed using a GSI-washout experiment (figure 3.B) which showed that Notch-induced CD28 upregulation is not affected by cyclohexamide and so does not require *de novo *protein synthesis. Finally, DN-MAML downregulated CD28 mRNA and cell surface expression (figure 3.C&3D), confirming the contribution of endogenous Notch to basal CD28 expression and also showing that the transcriptional activity of Notch is necessary for this effect. Together, the upregulation of CD28 in the absence of *de novo *protein synthesis and the requirement of the transcriptional activity of Notch shows that CD28 is a direct transcriptional target of Notch. This finding is in agreement with a recent study by Margolin *et al*. which used ChIP-on-chip to identify direct transcriptional targets of Notch1 [31] and found that there was a high degree of significance in the affinity of Notch1 for the CD28 promoter (see Additional file 4).

Finally, we transduced primary peripheral blood CD3+ T cells with GFP-alone, N1ΔE, or N1ΔE with GSIs, and then cells were stained for cell surface CD28. As shown in figure 3.E, a small increase in CD28 expression in response to Notch was observed in these cells, while GSI treatment reduced CD28 expression to below that of untreated cells. While these are preliminary findings, it is clear that Notch regulates CD28 expression in both cell lines and in primary cells.

### PCR Validation of Affymetrix Data using Ectopic Notch

**Figure 4:**
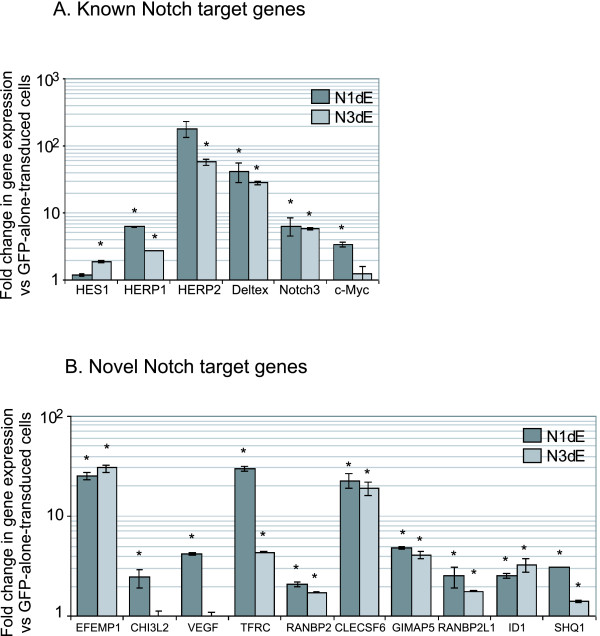
**PCR validation of Affymetrix microarray data**. cDNA from GFP-alone, N1ΔE, or N3ΔE-transduced Jurkat cells was used for PCR for a panel of known Notch target genes (A) as well as 10 novel genes most upregulated by Notch1 (B) based on microarray data in figure 1. Data represents fold change in gene expression from GFP-alone-transduced cells.

In order to validate this microarray data we used cDNA from N1ΔE and N3ΔE-transduced Jurkat cells. Real-time PCR analysis using a panel of known Notch target genes confirmed the presence of active Notch signalling in Notch-transduced cells (figure 4.A). These genes were HES1 [23], HERP1&2 [15], Deltex [32], Notch3 [24] and c-Myc [10]. Although the level of gene upregulation varied, there was a general pattern of upregulation of these genes in Notch-transduced cells.

We then sought to validate the expression of the 10 novel genes most up-regulated by Notch1: EGF-containing fibulin-like extracellular matrix protein 1 (EFEMP1 or fibilin3), chitinase 3-like 2 (CHI3L2), vascular endothelial growth factor (VEGF), transferrin receptor (TFRC), RAN binding protein 2 (RANBP2), C-type lectin, superfamily member 6 (CLECSF6), immune associated nucleotide 4 like 1 (IAN4L1 or GTPase, IMAP family member 5 (GIMAP5)), RAN binding protein 2-like 1 (RANBP2L1), inhibitor of DNA binding 1 (ID1), and SnoRNAs of the box H/ACA Quantitative accumulation (SHQ1). Real-time PCR analysis of cDNA from N1ΔE and N3ΔE-transduced Jurkat cells confirmed the upregulation of these genes in response to Notch signalling (figure 4.B). We further extensively validated this data using a panel of 6 T-ALL and non-T-ALL cell lines transduced with GFP-alone, N1ΔE or N3ΔE retrovirus. As shown in Additional file 5, data from these lines are broadly consistent with data from Jurkat cells. Overall, this PCR analysis of cells transduced with ectopic Notch has validated the Affymetrix data.

### Expression of Notch Target Genes following Inhibition of Notch Signalling

**Figure 5:**
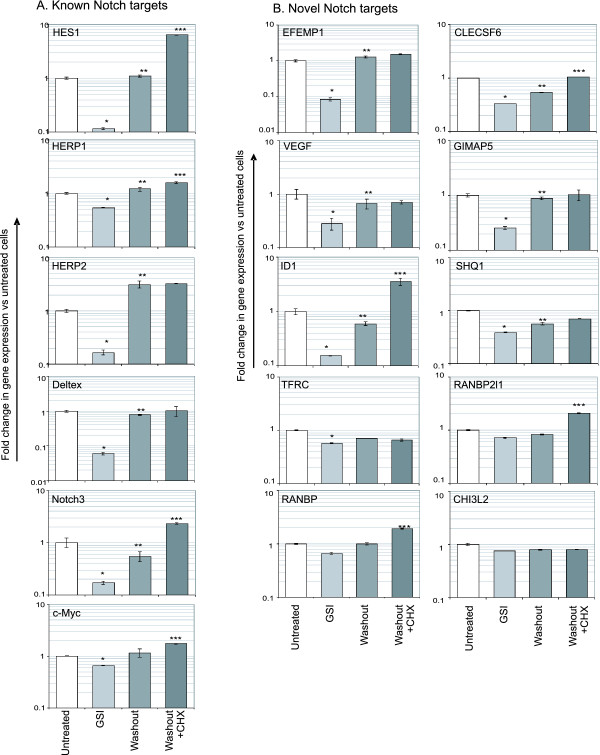
**Identification of direct notch targets using a GSI washout experiment**. Jurkat cells were treated with 10 uM GSI (or DMSO (untreated)) for 48 hours to accumulate cell surface Notch before washing to permit Notch signalling. After washing, cells were treated with 20 uM cyclohexamide (CHX) or ethanol (vehicle control) to inhibit protein synthesis. After 4 hrs, RNA was isolated and cDNA made for real-time PCR analysis of known Notch target genes (A) and novel Notch target genes (B). Expression values were calculated using cDNA from untreated cells as the calibrator sample. * represents p < 0.01 versus untreated cells (effect of Notch inhibition). ** prepresents p < 0.01 versus GSI-treated cells (effect of Notch signalling). *** represents p < 0.01 versus washout cells (effect of cyclohexamide treatment).

To investigate the response dynamics of the Notch target genes identified by Affymetrix microarray analysis, we used a GSI washout assay to measure gene expression in response to endogenous Notch signalling. This assay involves incubating cells with GSI to allow Notch to accumulate at the cell surface. Washing the cells then removes gamma secretase inhibition and leads to active Notch signalling [9]. As shown in figure 5.A, mRNA expression analysis of known Notch target genes confirmed the validity of this method by showing an increase in gene expression following the removal of gamma secretase inhibition. In all cases, GSI treatment led to a significant decrease in gene expression, although the inhibition of c-Myc expression was not as striking as other known Notch targets. As expected, expression of these known Notch target genes increased following GSI washout and in some cases, expression increased above that of the untreated cells. Some cells were also incubated in parallel with cyclohexamide to inhibit protein synthesis and allow us to determine if *de novo *protein synthesis is required for gene expression. This in turn would indicate whether these genes are direct or indirect Notch targets. As expected, cyclohexamide did not prevent the increased gene expression following GSI washout in known Notch target genes (which have mostly been characterised as direct transcriptional targets). On the contrary, there was a general increase in gene expression in the presence of cyclohexamide. One explanation for this could be that an inhibitor of gene expression (such as HES1) provides negative feedback for Notch target genes in normal circumstances. This is supported by the finding that HES1 physically interacts with CSL to inhibit Notch/CSL-mediated transcription [33]. Moreover, oscillations in HES1 expression have been found to be due to auto-inhibition of HES1 transcription [34]. In the presence of cyclohexamide, a reduction in the protein level of an inhibitor such as HES1 may allow Notch to increase gene expression levels without any negative feedback mechanism.

When novel Notch target genes were analysed (figure 5.B), we found a significant decrease in gene expression in all cases following GSI treatment, although the degree of reduction in gene expression for RANBP, RANBP2L1, TFRC and CHI3L2 was small compared to known Notch target genes. Of the other genes (EFEMP1, CLECSF6, GIMAP5, VEGF, ID1 and SHQ1), all showed a significant increase in gene expression 4 hrs following GSI washout, and this was not affected by the presence of cyclohexamide, indicating that *de novo *protein synthesis is not required for the Notch-induced transcription of these genes.

Inhibition of known and novel Notch target gene expression was also analysed by GSI treatment of a panel of 5 T-ALL cell lines. Following treatment with GSIs (10 uM GSI IX for 24 hrs), real-time PCR was used to measure reduction in target gene expression and the mean fold change from these 5 T-ALL lines are shown in Additional file 6. The data broadly correlates with those of the GSI washout experiment.

**Figure 6:**
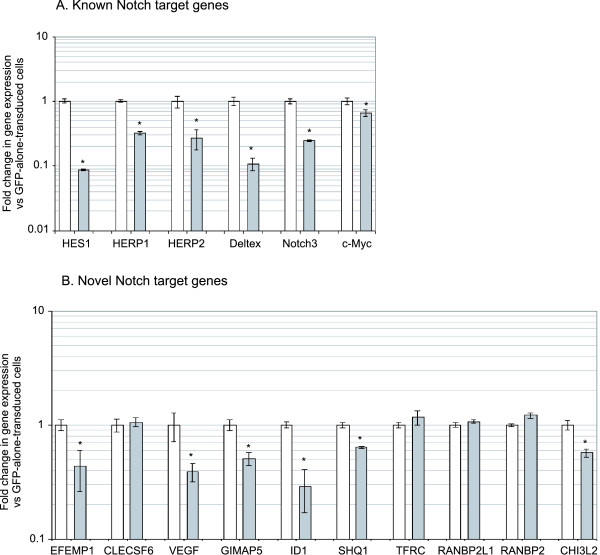
**Inhibition of Notch signalling using DN-MAML**. Jurkat cells were transduced with pMSCV (GFP-alone control vector) or DN-MAML and the gene expression levels of known Notch target genes (A) and novel Notch target genes (B) were analysed. * represents p < 0.01 versus the GFP-alone control cDNA sample.

Finally, we analysed gene expression in Jurkat cells transduced with GFP-alone vector or a dominant-negative mastermind-1 (DN-MAML) construct. This construct inhibits the transcriptional activity of Notch signalling and as can be seen in figure 6, inhibits the transcription of known Notch target genes. When novel Notch target genes were analysed, 4 genes out of 10 (TFRC, RANBP2, RANBP2L1 and CLECSF6) were not downregulated in the presence of DN-MAML.

Combining the data from figures 5B &6B, EFEMP1, VEGF, GIMAP5, ID1 and SHQ1, are upregulated by GSI-washout (without a requirement for *de novo *protein synthesis) and require Notch transcriptional activity since DN-MAML downregulates these genes, indicating that this set of genes are novel direct transcriptional targets of Notch. In support of this finding, Margolin *et al*. have recently performed a ChIP-on-chip study using T-ALL cell lines to identify direct transcriptional targets of Notch signalling. We have analysed the data from this study, focussing on the genes identified by us, and found that the genes whose promoter regions show significant Notch1 binding (EFEMP1, VEGF, IAN4L1/GIMAP5, ID1, SHQ1 and CD28; Additional file 4) are generally those which respond significantly in the GSI washout experiment (figure 5.B).

### Genes Downregulated by Notch

**Figure 7:**
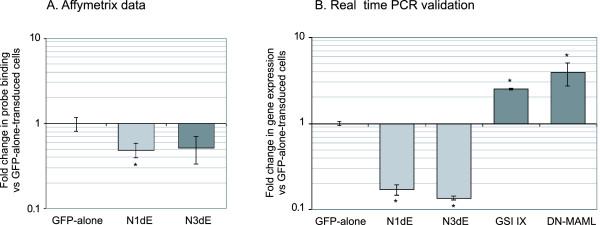
**IGLL1 is downregulated by Notch signalling in Jurkat cells**. (A) Affymetrix data from Notch-transduced Jurkat cells. (B) cDNA from GFP-alone, N1ΔE, or N3ΔE-transduced Jurkat cells was used for PCR analysis of IGLL1 expression (using GFP-alone-treated cells as the calibrator). IGLL1 expression in response to GSI treatment was also analysed using DMSO (vehicle control)-treated cells as the calibrator. Expression of IGLL1 in DN-MAML-expressing cells was analysed using pMSCV (GFP-alone vector)-transduced cells as the calibrator. * represents p < 0.05 vesus the relevant calibrator sample.

We also investigated genes downregulated by Notch signalling. It is likely that such genes are secondary targets of Notch whose transcription is inhibited by bHLH repressors such as HES1, HERP1&2 or ID1. However, real-time PCR analysis of cDNA from T-ALL cells failed to validate the majority of genes identified by microarray analysis as downregulated by Notch. One exception was IGLL1 (CD179b; figure 7), where ectopic Notch down-regulates IGLL1 expression, while GSI-treatment or DNMAML expression increases IGLL1 expression in Jurkat cells. However this effect was not consistently seen in other T-ALL cell lines. Mutations in IGLL1 have been shown to lead to B cell deficiencies in both mice and humans ([35]) and given the role of Notch in promoting T cell development at the expense of B cell fate, it is possible that one such mechanism could be the downregulation of IGLL1.

### VEGF, ID1 and GIMAP5 are upregulated by Notch at the protein level

Of the novel Notch target genes so far analysed at the mRNA level, we chose to focus on VEGF, ID1, and GIMAP5 because of their known involvement in cancer or T cell development.

**Figure 8:**
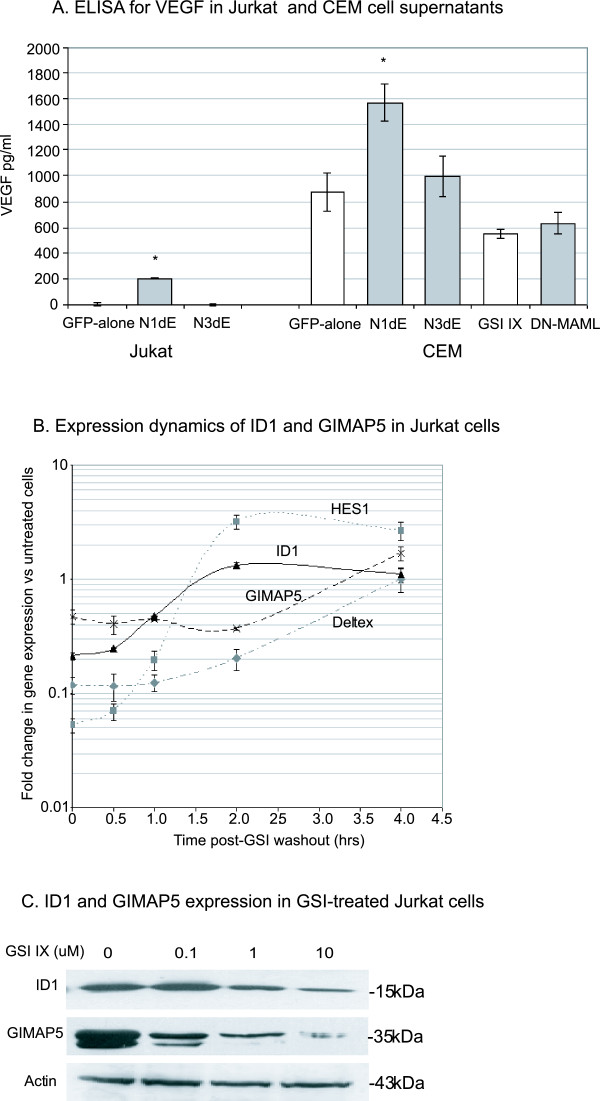
**Analysis of Notch-induced VEGF, ID1 and GIMAP5 expression**. (A) Supernatants from GFP-alone, N1ΔE, or N3ΔE-transduced Jurkat or CEM cells were used for VEGF ELISAs to analyse secreted VEGF protein levels in response to ectopic Notch. Supernatants of CEM cells transduced with DN-MAML were also analysed to show the contribution of endogenous Notch transcriptional activity on VEGF expression. * represents p < 0.05 versus GFP-alone-transduced cells (B) Jurkat cells were used for a GSI washout assay and ID1 and GIMAP5 gene expression analysed over 4 hrs following washout using cDNA from untreated cells as the calibrator sample. (C) Western blot analysis of ID1 and GIMAP5 expression using protein extracts from Jurkat cells treated with a dose range of GSI IX.

At the mRNA level, VEGF is expressed at low levels in GFP-alone transfected Jurkat cells and is only upregulated by ectopic Notch1 (not Notch3). To confirm this finding at the protein level, we performed ELISAs on supernatants of cells transduced with GFP-alone, N1ΔE and N3ΔE retroviruses. As can be seen in figure 8.A, virtually no basal expression of VEGF protein is detected in supernatants from GFP-alone or N3ΔE-transduced Jurkat cells, whereas N1ΔE-transduced cells produce detectable levels of VEGF. The lack of detectable basal levels of secreted VEGF protein is contrary to the gene expression data shown in figures 5 &6, where GSI treatment and expression of DN-MAML decreased VEGF mRNA levels in Jurkat cells. This lack of correlation between VEGF mRNA and secreted VEGF protein levels could be due to a number of factors including post-transcriptional regulation of VEGF expression or regulation of VEGF protein secretion in the cell supernatants. This finding suggests that although ectopic Notch1 may promote VEGF protein expression, Notch does not necessarily contribute to basal VEGF protein expression in T-ALL cells.

We next analysed CEM cells which express detectable levels of secreted VEGF protein (figure 8.A). As with Jurkat cells, ectopic expression of Notch1, but not Notch3 upregulated VEGF protein expression. However, transduction with DN-MAML did not downregulate VEGF protein expression. Overall, these findings confirm the upregulation of VEGF by ectopic Notch1, however it is unlikely that endogenous Notch regulates basal VEGF expression in T-ALL cells.

ID1 expression was found to be upregulated by both Notch1 and Notch3 (figure 4.A) and downregulated by GSI-treatment and DN-MAML (figures 5 &6). Analysis of gene expression in Jurkat cells following GSI washout showed a rapid induction of ID1 transcription (within a similar time-scale to that of HES1 and Deltex; figure 8.B). GSI-dependent downregulation of ID1 protein expression was confirmed by western blotting (figure 8.C), confirming that endogenous Notch signalling regulates ID1 expression.

GIMAP5 (IAN4L1) belongs to a family of signalling proteins which are thought to regulate T cell development and survival [36]. GIMAP5 has been shown to have anti-apoptotic functions and has been shown to physically interact with Bcl-2 [37,38]. As such it represents a good candidate protein for mediating the anti-apoptotic functions of Notch1. Induction of gene expression occurred within 4 hrs of GSI-washout (figure 8.B), and regulation by Notch at the protein level was confirmed by Western blotting (figure 8.C). Furthermore, in a separate study we have used siRNA-mediated knockdown of GIMAP5 expression to show that GIMAP5 mediates some of the protective effect of Notch to glucocorticoid-induced apoptosis (manuscript submitted for publication).

Other members of the GIMAP family are not represented on the Affymetrix array, so we sought to determine whether these genes, like GIMAP5, are also regulated by Notch. As shown in Additional file 7, a general upregulation of all GIMAP family genes (GIMAP3 being a pseudogene) in response to either ectopic Notch1 or Notch3 is seen in Jurkat cells. Furthermore, in a sample of primary CD3+ preripheral blood cells, ectopic Notch1 generally upregulated this family of genes, while GSI-treatment reduced their expression levels (Additional file 7). These data indicate that the GIMAP family of proteins may be key mediators of Notch-induced regulation of T-cell development.

## Discussion

In this study we have used an approach utilising ectopic expression of Notch to identify novel target genes in T-ALL. Using this approach we have identified a set of novel Notch target genes and we validated the Affymetrix microarray data by real-time PCR analysis of the top 10 novel Notch1 target genes using a panel of cell lines transduced with Notch constructs. Although we have found little overlap between our set of Notch targets and those of other studies where Notch target genes have been identified by GSI treatment [9,10,39,40], some genes have been identified previously: SHQ1 [10], VEGF and ID1 [40].

This relative lack of overlap between our study and those of others probably reflects the different approaches taken by us (ectopic expression of Notch constructs) and others (GSI-inhibition of endogenous Notch). It is possible that Notch targets expressed at a low level endogenously may be more clearly identified in a microarray following ectopic Notch expression, whereas targets expressed at saturating levels may not be further upregulated by ectopic Notch (and not easily identified by microarray analysis) but may be more readily identified by inhibition of endogenous Notch activity. The level of Notch activity in Jurkat cells (and other T-ALL cells used in this study) is clearly not saturated since many known Notch target genes are upregulated following ectopic Notch expression (based on microarray and PCR analysis), suggesting that this approach is a valid way of identifying novel targets of Notch signalling. Furthermore, given the cell context-specificity of Notch target gene expression, it was important for us to use a T-ALL cell line in our study which has aimed to identify such relevant to T cell leukemia, even though Jurkat cells already express an overactive form of Notch1. It is possible that a combined approach of overexpression and knockdown could reveal a more complete set of target genes following microarray analysis.

To determine which of our set of putative Notch target genes are regulated by endogenous Notch signals we used GSI-mediated inhibition of Notch activation, and DN-MAML-mediated inhibition of Notch transcriptional activity. This strategy showed the majority of these genes to be regulated by endogenous Notch activity.

Recently, Margolin et al. have performed a genome-wide ChIP-on-chip study to identify direct transcriptional targets of Notch1 [31]. Probe binding affinities to Notch/CSL/DNA complexes were ranked in order of p-values in order to identify significant physical interactions between Notch1 and gene promoters. Analysis of this data has confirmed that several of the genes identified by us are direct targets of Notch signalling (EFEMP1, VEGF, ID1, SHQ1, IAN4L1/GIMAP5, CD28).

Of the 10 genes most upregulated by Notch1, we found four to be of particular interest: VEGF, ID1, IAN4L1 (GIMAP5), and CD28. At the protein level, VEGF was shown to be upregulated by Notch1 (but not Notch3) in Jurkat and CEM cells, although VEGF expression was not downregulated by either GSI treatment or DN-MAML. This finding was notable since with the exception of VEGF transcriptional differences between Notch1 and Notch3 were limited to the extent of gene regulation, an unsurprising finding given that all Notch homologues mediate transcription via CSL. The fact that ectopic Notch1 but not ectopic Notch3 can upregulate VEGF expression may indicate the presence of a mechanism whereby Notch1 may interact with factors upstream of VEGF expression in a gamma secretase-independent fashion. VEGF has previously been shown to be expressed by T-ALL cell lines [41,42] and may contribute to angiogenesis in T cell lymphomas. As such, Notch-induced VEGF expression may represent an important step in lymphoma development.

ID1 expression was also found to be induced by Notch and the identification of this gene as a transcriptional target of Notch is not surprising given that ID1 belongs to the same family of basic helix-loop-helix proteins as HES1 and HERP1&2 [43]. Two studies have shown have also shown ID1 to be downstream of Notch signalling: Talora *et al*. [44] have shown that Notch3 transgenic mice express high ID1 levels, and that Notch induced ID1 expression is mediated by pre-TCR-induced extracellular-signalling-regulated kinase 1/2. Secondly, Fox et al. [45] have shown an increase in ID1 expression in human embryonic stem cells transfected with Notch. Our data now shows that Notch regulates ID1 expression in T-ALL cell lines.

GIMAP5 was found to be upregulated by Notch and, whilst the exact role of GIMAP5 is unclear, it has been shown to interact with Bcl-family members and play an important role in inhibiting apoptosis during T cell development [36]. Further studies will determine the role of GIMAP5 in mediating the functional effects of Notch during normal thymocyte development and in the development of T cell leukaemia. We have investigated the relationship between GIMAP5 upregulation and apoptosis in T-ALL cells (manuscript submitted for publication).

Our finding that CD28 is a direct target of Notch signalling is of interest both in terms of T cells development and leukaemia, and also in mature T cell activation. The role of CD28 in T cell development is unclear. CD28 stimulation in developing thymocytes has been shown to be important for regulatory T cell development [46], as has Notch signalling [47], and it is therefore possible that Notch-induced CD28 expression may mediate this developmental process. The role of CD28 in thymocyte apoptosis is unclear. CD28 activation can inhibit glucocorticoid-mediated apoptosis ((([26,27,30], however other studies show a pro-apoptotic role of CD28 stimulation during negative selection (e.g. [28,29]) that is determined by signal strength [48]. It is clear from our experiments that although Notch signalling regulates CD28 expression, CD28 expression is not solely dependent on Notch signalling since neither GSI treatment, nor DN-MAML, abolishes CD28 expression. It is likely that Notch signalling plays a role in "fine-tuning" CD28 expression and thus helping to determine the fate of developing thymocytes. Although we have shown that Notch can regulate CD28 expression in peripheral blood T cells, it remains to be seen whether Notch is able to regulate CD28 expression in primary thymocytes.

## Conclusion

We have identified novel transcriptional targets of Notch signalling in T cell leukaemia, and confirmed changes at the protein level for several of these targets which have a known role in cancer and T cell development. The identification of these genes will form the basis of further studies aimed at understanding the mechanism of Notch induced changes in T-ALL cells.

## Competing interests

The authors declare that they have no competing interests.

## Authors' contributions

NC performed the experimental work with help from VP, CF, FW and SH. LZ performed the bioinformatics on the microarray data. NC and AMB conceived of the study, and participated in its design and coordination and helped to draft the manuscript. All authors read and approved the final manuscript.

## Supplementary Material

Additional file 1 **PCR primer sequences**. Sequences of PCR primers used to detect expression of known and novel Notch target genes. Click here for file

Additional file 2 **Notch expression in T-ALL cells**. (A) cDNA from parental Jurkat CEM and Molt4 cells was analysed for the expression of Notch homologues. Quantitative expression was determined using a standard made up of known numbers of copies of human genomic DNA. (B) Luciferase assay analysis of CSL-Luciferase reporter activity in HEK294T cells transfected with Notch ICD or ΔE constructs. Click here for file

Additional file 3 **Affymetrix microarray analysis of Notch3-transduced Jurkat cells**. Jurkat cells were transduced with GFP-alone, N1ΔE, or N3ΔE retrovirus and after 48 hrs, GFP+ cells were isolated by flow cytometry and total RNA extracted for Affymetrix array analysis. Data presented represents mean of 4 independent experiments. Graphical representation of microarray data showing the 15 genes most upregulated by Notch3. * represents p < 0.05 vesus GFP-alone-transduced cells based on p-values from n = 4 experiments. Click here for file

Additional file 4 **ChIP-on-chip data from Margolin et al. PNAS 2009**. Raw data from the Margolin et al. PNAS 2009 study was used to determine the ChIP-on-chip significance of Notch1 biding to the promoter regions of the genes identified in this study. p-values < 0.05 are shown in bold. Click here for file

Additional file 5 **Expression of novel Notch target genes in cell lines transduced with Notch**. cDNA from GFP-alone pMX, N1ΔE, or N3ΔE-transduced T-ALL and non-T-ALL cell lines wwere used for PCR analysis of the 10 novel genes most upregulated by Notch1 based on microarray data in figure 1. Data represents fold change in gene expression from GFP-alone-transduced cells. Click here for file

Additional file 6 **Gene expression following GSI treatment of T-ALL cell lines**. 5 T-ALL cell lines (Jurkat, CEM, Molt4, HPB-ALL and SIL-ALL) were treated with DMSO or 10 uM GSI IX for 24 hrs and cDNA from these cells used for PCR analysis of Notch target genes. Fold change in gene expression (compared to DMSO-treated cells) was used to determine mean fold change in up to 5 T-ALL cell lines (where target genes were expressed). p-values < 0.05 are shown in bold. Click here for file

Additional file 7 **Upregulation of the GIMAP gene family by Notch**. (A) cDNA from Jurkat cells transduced with GFP-alone, N1ΔE or N3ΔE retrovirus was used for real-time PCR analysis of GIMAP gene expression. GIMAP3 is a pseudogene. GIMAP8 was not expressed in Jurkat cells. * represents p < 0.05 vesus the GFP-alone-transduced sample. Data for GIMAP5 is shown in figure 4.B. (B) cDNA from primary CD3+ peripheral blood cells transduced with GFP-alone, N1ΔE or N1ΔE+GSIs was used for PCR analysis of GIMAP5 family gene expression. GIMAP7 was not expressed in these cells. Click here for file
